# Mitochondrial cristae remodeling in ischemic stroke: mechanisms and therapeutic perspectives

**DOI:** 10.3389/fphar.2026.2017406

**Published:** 2026-10-26

**Authors:** Dawei Zhang, Hongbo Zhang, Ruoxin Tu, Shuo Zhang, Hao Tian, Yue Meng, Xiaolin Lv, Hongna Yin

**Affiliations:** 1 Heilongjiang University of Chinese Medicine, Harbin, China; 2 The Second Affiliated Hospital of Heilongjiang University of Chinese Medicine, Harbin, China

**Keywords:** cardiolipin, ischemic stroke, mitochondrial cristae, neuroinflammation, OPA1

## Abstract

Mitochondrial cristae arrange the respiratory machinery within the inner membrane and change as ischemia gives way to reperfusion. Their organization depends on mitochondrial contact site and cristae organizing system assemblies, optic atrophy 1, adenosine triphosphate synthase, and cardiolipin. Disruption of these systems can impair energy production, facilitate neuronal death, and expose mitochondrial DNA to inflammatory sensors. This review brings structural work together with experimental stroke studies to examine how crista remodeling contributes to injury and recovery. Genetic manipulation of selected regulators has preserved mitochondrial structure and reduced injury in neurons and microglia. Most of these interventions preceded ischemia, leaving the speed and feasibility of repair after stroke onset uncertain. Pharmacological modulation and mitochondrial delivery offer ways to address this gap, particularly around reperfusion. Emerging strategies combine modulation of crista protein assemblies with cardiolipin directed treatment, mitochondrial antioxidant nanomedicine, organelle delivery, and control of local drug exposure. Their therapeutic evaluation can be aligned with distinct opportunities at reperfusion and during subsequent tissue recovery. Progress toward treatment will require evidence that a candidate reaches the affected brain cells, modifies the intended membrane assembly, and produces lasting neurological benefit. The available studies place crista remodeling within neuroprotective pathways and identify the experimental steps needed to evaluate it as a therapeutic target.

## 1 Introduction

Stroke remains a major cause of death and disability. Ischemic events accounted for approximately two-thirds of new strokes worldwide in 2021 (GBD, 2021 Stroke Risk Factor Collaborators, 2024). Thrombectomy has improved outcomes even among selected patients with large infarcts, although substantial disability remains common after treatment (Costalat et al., 2024). Adjunctive neuroprotection has been less consistent. Nerinetide did not improve the primary functional outcome in ESCAPE-NEXT, a randomized trial conducted alongside thrombectomy (Hill et al., 2025). The continuing disability after reperfusion motivates efforts to preserve cellular function as oxygen and substrates return to injured tissue.

Oxidative phosphorylation (OXPHOS) takes place within the mitochondrial inner membrane. Cristae organize its respiratory proteins into distinct domains and create compartments within the organelle. This arrangement is exposed to changing stresses during oxygen deprivation and reoxygenation. In diabetic rats, electron microscopy showed mitochondrial swelling and crista disruption that evolved over several reperfusion intervals (Wang and Chen, 2025). The persistence of structural injury after flow restoration makes membrane recovery relevant to the timing of neuroprotection.

The location of energy-converting proteins is closely related to crista curvature. *In situ* cryo-electron tomography of *Polytomella* mitochondria revealed helical rows of adenosine triphosphate (ATP) synthase along crista ridges (Dietrich et al., 2024). Native imaging in *Chlamydomonas* placed ATP synthase at curved edges and respiratory assemblies in flatter membrane regions (Waltz et al., 2025). These algal structures resolve a spatial arrangement that can guide investigation of mammalian cristae.

Mammalian cristae change with protein processing and tissue age. Altering the balance of long and short optic atrophy 1 (OPA1) isoforms changed crista packing and respiration in mouse embryonic fibroblasts (Fry et al., 2024). Aging human and mouse hearts had lower crista density and oxidative capacity despite relatively preserved abundance of several respiratory components (Molina-Riquelme et al., 2026). Protein abundance captures only part of this remodeling, since the same components can occupy different membrane arrangements.

Stroke models connect crista organization with tissue injury. Increasing sorting and assembly machinery component 50 (SAMM50) improved mitochondrial ultrastructure and neurological outcomes in rats (Yin et al., 2022). Membrane disorganization may also extend the response beyond energy failure. In a mitochondrial genetic screen, loss of several crista organizers increased mitochondrial DNA (mtDNA) release and interferon activation in cultured cells (He et al., 2022). The relationship between architecture, respiration, and inflammatory signaling places crista remodeling at the intersection of several injury pathways.

This review examines the molecular assemblies that shape cristae, their contribution to ischemic injury, and approaches to therapeutic modulation. Structural evidence is considered alongside experiments in which manipulation of a regulator changes mitochondrial architecture and stroke outcomes. The final sections address treatment timing, target engagement, and the evidence needed to move from protective genetic interventions to therapy after onset.

## 2 Molecular control of cristae architecture

Cristae contain narrow junctions, broad membrane surfaces, and highly curved edges. Native tomography places ATP synthase preferentially at the edges and respiratory assemblies in adjoining flatter regions (Waltz et al., 2025). The following sections consider the scaffolds, remodeling proteins, and lipids that maintain this organization and respond to mitochondrial stress (Figure 1).

**FIGURE 1:**
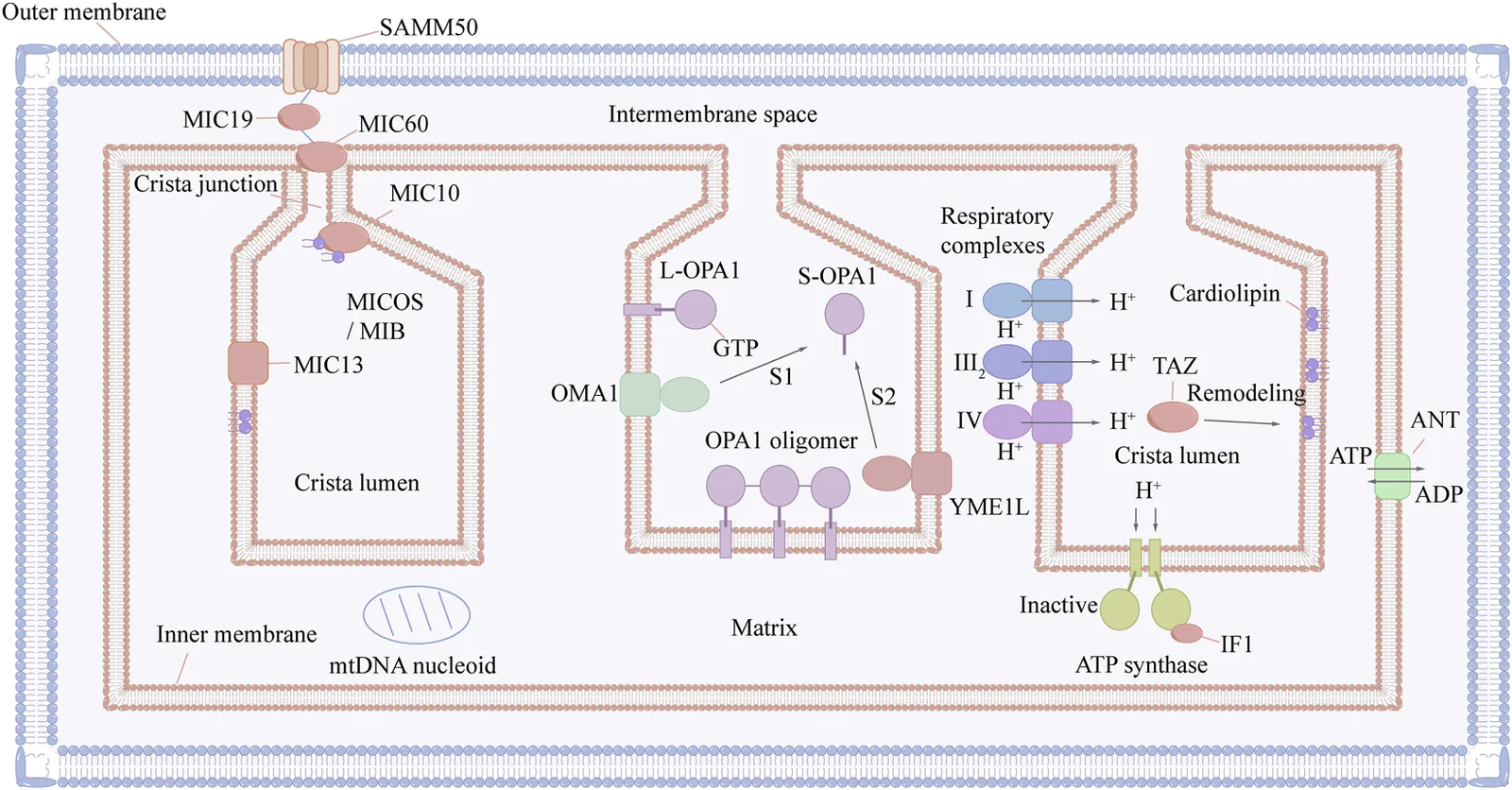
Molecular organization and regulation of mitochondrial cristae. The mitochondrial inner membrane forms cristae whose lumens communicate with the intermembrane space through narrow crista junctions. The mitochondrial contact site and cristae organizing system (MICOS), including MIC60, MIC19, MIC10, and MIC13, supports junction formation and membrane organization. The SAMM50–MIC19–MIC60 connection forms part of the mitochondrial intermembrane bridge (MIB), linking the outer and inner membranes (Tang et al., 2020; Bock-Bierbaum et al., 2022). Membrane-anchored long OPA1 (L-OPA1) and proteolytically generated short OPA1 (S-OPA1) participate in GTP-dependent membrane remodeling. OMA1 and YME1L cleave OPA1 at the S1 and S2 sites, respectively; additional processing sites are omitted for clarity (Wang et al., 2021). Respiratory complexes I, III_2_, and IV pump protons from the matrix into the crista lumen. ATP synthase dimers contribute to crista curvature, and proton return through active ATP synthase supports ATP production. The IF1-associated enzyme depicts an inhibited ATP synthase population (Romero-Carramiñana et al., 2023). Tafazzin-mediated cardiolipin remodeling supports the lipid environment of inner-membrane proteins, while ANT exchanges matrix ATP for extramitochondrial ADP. Mitochondrial DNA nucleoids reside in the matrix. Arrows indicate the depicted processing reactions, transport directions, or functional relationships. The schematic is not drawn to scale. Abbreviations: ADP, adenosine diphosphate; ANT, adenine nucleotide translocase; ATP, adenosine triphosphate; GTP, guanosine triphosphate; H^+^, proton; IF1, ATP synthase inhibitory factor 1; MIC10/13/19/60, MICOS subunits 10/13/19/60; mtDNA, mitochondrial DNA; OMA1, mitochondrial stress-activated metalloprotease; OPA1, optic atrophy 1; SAMM50, sorting and assembly machinery component 50; TAZ, tafazzin; YME1L, YME1-like 1 ATPase.

### 2.1 MICOS and MIB assembly

The outer and inner mitochondrial membranes are separate phospholipid bilayers. Invaginations of the inner membrane form cristae whose lumens remain connected to the intermembrane space through narrow junctions. The mitochondrial contact site and cristae organizing system (MICOS) establishes these junctions. MICOS complex subunit 60 (MIC60) and MICOS complex subunit 19 (MIC19) form its central scaffold. MIC19 promotes MIC60 tetramerization, producing a curved assembly suited to the junction. The central coiled-coil domain of MIC60 forms an elongated, bow tie-shaped tetramer. Interaction with MIC19 stabilizes this arrangement, while dimerization of the mitofilin domains exposes a crescent-shaped membrane-binding surface. Its curvature fits the neck of the crista junction, suggesting that the complex can act as a strut across the opening (Bock-Bierbaum et al., 2022). Integrative structures and molecular simulations of human MIC60 suggest an additional function as a diffusion barrier between the intracristal and intermembrane spaces (Nathanail et al., 2026). Junction geometry and molecular exchange may be coupled at the same scaffold.

MICOS complex subunit 10 (MIC10) contributes to curvature through a second subcomplex. Its positively charged surfaces recruit cardiolipin (CL) and favor oligomerization at curved junctions in simulations and model membranes. Conserved positive loop motifs concentrate CL around the proteins, linking local lipid enrichment to subcomplex assembly. Multiscale simulations reconstructed this network in a realistic junction geometry and found that it could maintain membrane bending. Disordered regions were proposed to contribute an additional barrier to exchange across the junction. This model places lipid recruitment, curvature, and permeability within one assembly, with their coupling still to be tested under ischemic stress (Brown et al., 2026). Assembly is regulated by protein turnover. MICOS complex subunit 13 (MIC13) and stomatin-like protein 2 (SLP2) coordinate the MIC10 and MIC60 subcomplexes. In human knockout cells, depletion of YME1-like 1 ATPase (YME1L1) stabilized the MIC10 subcomplex and partly restored junctions in the absence of MIC13. SLP2 protected MICOS complex subunit 26 (MIC26) from degradation in the same network (Naha et al., 2024). MIC10 also supported ATP synthase oligomerization independently of some MICOS functions in yeast, extending its influence from junctions to the crista edge (Rampelt et al., 2022).

The junction scaffold is connected to the outer membrane through SAMM50 and MIC19. This mitochondrial intermembrane bridge (MIB) maintains contacts between the two membranes. Caspase recruitment domain family member 19 (CARD19) associated with MIB components in macrophages, where its loss accompanied irregular cristae (Rios et al., 2022). In hepatocytes, MIC19 deletion affected crista structure, lipid metabolism, and contacts with the endoplasmic reticulum (Dong et al., 2024). MIB organization sits within a network that links crista junctions to the surrounding membranes and their lipid environment.

Stress-dependent proteolysis changes the continuity of the bridge. Overlapping activity with m-AAA protease 1 (OMA1) cleaves the amino terminus of MIC19 and disrupts the SAMM50-MIC19-MIC60 connection. In cells, this cleavage accompanied fewer junctions and lower ATP production (Tang et al., 2020). OMA1 also associated with MIC60 independently of OPA1. OMA1-null cells lost some MICOS contacts and bioenergetic responses, which an artificial membrane tether partly restored (Viana et al., 2021). The protease has both disruptive and supportive architectural functions. Its effects extend to the processing of OPA1, another regulator of the inner membrane.

### 2.2 OPA1-dependent cristae remodeling

OPA1 binds and reshapes the inner membrane through guanosine triphosphate (GTP)-dependent oligomerization. Human OPA1 structures identified a membrane-inserting loop within the paddle domain and interfaces that support assembly on CL-rich membranes. Insertion of the loop into the bilayer stabilizes this association. Dimerization through the paddle domain supports a flexible helical lattice along the membrane. Conformational changes can withdraw the inserting loop from the outer leaflet, offering a physical route for remodeling the bilayer. Mutating these contacts changed mitochondrial morphology in cells, linking the resolved interfaces to protein function (von der Malsburg et al., 2023). On OPA1-coated lipid tubes, nucleotide-dependent pairing of GTPase domains was associated with different helical arrangements. The assemblies formed closely packed protein rungs with limited connections between adjacent rungs. Mutations affecting assembly interfaces or membrane binding produced fragmentation in cell-based assays. These results connect the structural behavior of purified OPA1 with its ability to maintain mitochondrial organization in cells (Nyenhuis et al., 2023). Lipid binding and nucleotide state jointly influence the geometry of the OPA1 assembly.

Membrane-anchored long OPA1 (L-OPA1) and proteolytically generated short OPA1 (S-OPA1) make distinct contributions to crista organization. Cryo-focused ion beam milling and *in situ* tomography provided three-dimensional views of fibroblasts with defined isoform states. Increased L-OPA1 favored stacked cristae and elongated mitochondria, whereas a greater S-OPA1 contribution correlated with irregular packing and rounder organelles. Functional assays detected compromised respiration under isoform imbalance and changes in calcium handling. The comparison indicates that junction organization, membrane packing, and overall organelle shape respond differently to the available OPA1 forms (Fry et al., 2024). Transcript choice determines which cleavage sites are available. Cells lacking S2-containing transcripts revealed an additional YME1L1-dependent S3 site whose products influenced mitochondrial fusion (Wang et al., 2021). Processing also supports adaptation under some forms of stress. Noncleavable OPA1 supported mouse development, while OPA1 processing improved survival during OXPHOS deficiency in a cardiomyopathy model (Ahola et al., 2024). The consequences of retaining an isoform depend on the energetic condition in which it functions.

OMA1 responds to disturbances in membrane potential. Brief membrane flickering in mammalian cells required copper transport by solute carrier family 25 member 3 (SLC25A3) and promoted OMA1 processing of OPA1 when fusion became excessive (Murata et al., 2024). After depolarization, OMA1 also clears stalled import substrates from mitochondrial protein channels (Krakowczyk et al., 2024). In coiled-coil-helix-coiled-coil-helix domain containing 10 (CHCHD10)-associated myopathy, OMA1 supported survival through local remodeling and a distal integrated stress response (Shammas et al., 2022). These functions make OPA1 cleavage part of a broader response that includes protein import and cellular stress signaling.

Posttranslational control adds a rapid regulatory layer beyond OPA1 abundance and cleavage, as SIRT3 mediated deacetylation increases OPA1 activity (Chen et al., 2024) and cysteine oxidation changes its oligomeric state and susceptibility to oxidative injury (Semenzato et al., 2023). Phosphorylation can stabilize MIC60 oligomers (Tsai et al., 2018) or alter coupling to PINK1 and Parkin dependent ubiquitin signaling (Akabane et al., 2016), providing routes for assembly remodeling and mitochondrial quality control to respond to energetic stress without awaiting new protein synthesis.

OPA1-dependent changes extend beyond membrane shape. Patient-associated mutations produced distinct crista defects, and some cells retained elongated mitochondria despite impaired fusion (Cartes-Saavedra et al., 2023). Depletion, overexpression, and selected disease variants changed mitochondrial nucleoid abundance or distribution (Macuada et al., 2024). OPA1 loss in fibroblasts altered endoplasmic reticulum-mitochondrial spacing and the calcium signal available for matrix uptake (Cartes-Saavedra et al., 2021). In hypothalamic proopiomelanocortin neurons, conditional *Opa1* deletion changed crista topology and calcium-dependent hormonal output (Gómez-Valadés et al., 2021). Genome organization and calcium handling are relevant accompanying responses when OPA1 is manipulated to protect neurons.

The capacity for OPA1-dependent remodeling varies with cell state. Aging human and mouse hearts showed reduced crista density and maximal respiration in association with OPA1 decline (Molina-Riquelme et al., 2026). YME1L1 deletion altered metabolism in adult neural stem cells and depleted the self-renewing pool (Wani et al., 2022). Modulating this processing network during stroke recovery may affect longer-term neural maintenance as well as the immediate response to ischemia.

### 2.3 ATP synthase organization and cardiolipin

ATP synthase dimers shape crista rims through the angle and arrangement of their monomers. Purified bovine dimers displayed a flexible interface (Spikes et al., 2021), and tomography in yeast and nematodes associated different dimer angles with distinct crista morphologies (Buzzard et al., 2024). In reconstituted lipid tubes, rotary activity favored sorting toward curved membranes and promoted transient monomer contacts (Valdivieso González et al., 2023). A planar dimer configuration can be linked by a straight ATP synthase inhibitory factor 1 (IF1) (Jiko et al., 2026). Native mammalian membranes contain several oligomeric arrangements (Nakano et al., 2026). The abundance of dimers is one component of a geometry that also changes with interface flexibility and enzyme activity.

ATP synthase assembly and catalytic activity can respond differently to the same perturbation. RNA interference against ATP synthase membrane subunit f (ATP5MF) in HeLa cells destabilized dimers and altered crista morphology. The amounts of other enzyme subunits and measured synthetic and hydrolytic activity remained relatively preserved. Re-expression of the f subunit restored mitochondrial morphology and permeability transition-dependent swelling. This rescue linked the structural phenotype to a defined subunit and showed that an abnormal crista arrangement can occur without a large change in measured bulk enzyme activity (Galber et al., 2021). IF1 generated an inactive enzyme pool enriched in oligomeric assemblies and high-potential crista-tip regions in mouse cells and tissues (Romero-Carramiñana et al., 2023). Removing IF1 from mouse intestinal epithelium reduced oligomerization and increased ATP turnover, accompanied by adenosine accumulation and inflammatory changes (Domínguez-Zorita et al., 2023). IF1 binding and the local electrochemical conditions help determine the energetic consequences of retaining an ATP synthase assembly.

CL, an anionic phospholipid enriched in the inner membrane, provides a lipid environment for crista-shaping and respiratory proteins. Protein crowding influences CL remodeling by tafazzin (TAZ), while defects in CL synthesis or remodeling reduce the density of OXPHOS proteins in liposome studies and tissue analyses (Xu et al., 2021). The resulting acyl composition affects membrane behavior. Increasing lipid saturation triggered an abrupt topological change in engineered yeast, and CL buffered the loss of curvature despite preserved ATP synthase dimerization. Experimental lipidome dissection was combined with a continuum model that incorporated lipid- and protein-mediated curvature. Small changes in membrane properties could move the system across a mechanical instability and collapse the folded topology. CL became particularly important under low-oxygen conditions that favored phospholipid saturation. This behavior offers a possible explanation for nonlinear structural responses to a gradually changing lipid environment (Venkatraman et al., 2023). Simulations of mammalian dimers predict that CL acyl chains can also alter dimer geometry (Makowski et al., 2025). Cell experiments with saturated CL found greater membrane rigidity and disruption of inner-membrane architecture (Venkatraman et al., 2026).

CL binding also stabilizes assemblies and supports nucleotide exchange. Cardiomyocyte-specific TAZ deletion changed CL quantity and species, disrupted MICOS and ATP synthase organization, and lowered respiratory capacity (Zhu et al., 2021). Distinct CL-binding sites on the adenine nucleotide translocase (ANT) contributed differently to carrier stability and transport. Biochemical assays, native mass spectrometry, and simulations examined the three tightly bound CL molecules within the carrier. Negatively charged substitutions disrupted the lipid interactions at individual sites. All tested sites contributed to tertiary stability, but transport was impaired to different extents depending on the site. A disease-associated mutation in the human carrier also compromised structure and nucleotide transport. The findings connect a local lipid-protein interaction with the exchange needed to sustain OXPHOS (Senoo et al., 2024). In CL-deficient yeast, phosphatidylglycerol partly occupied CL-binding positions within respiratory supercomplexes but changed their contacts and distribution (Hryc et al., 2023). Curvature, assembly stability, and transport provide several routes through which lipid remodeling can influence energy production.

Respiratory proteins form a range of assemblies within the crista membrane. *In situ* porcine structures resolved multiple supercomplex arrangements and their protein-lipid interfaces (Zheng et al., 2024). Mouse-tissue cryo-electron microscopy resolved selected respirasomes containing supercomplex assembly factor 1 (SCAF1) alongside a canonical composition without this factor (Vercellino and Sazanov, 2024). Native human structures showed that complex IV can complete late maturation after incorporation into a respirasome (Nguyen et al., 2026). Supercomplex abundance reflects a changing assembly process that includes enzyme maturation and selective stabilization.

The functional value of a respirasome depends on the respiratory defect. Mice with markedly reduced respirasome abundance retained respiratory capacity and normal exercise performance under the conditions tested (Milenkovic et al., 2023). Partial impairment of respiratory-complex biogenesis in flies increased stable supercomplexes without a parallel rise in respiration (Brischigliaro et al., 2023). In contrast, an enlarged complex I and III assembly compensated for complex III deficiency and limited cardiac ischemic injury in mice (Liang et al., 2025). Assembly remodeling can relieve a specific respiratory limitation, a property relevant to the changing substrate supply after cerebral reperfusion.

## 3 Cristae remodeling in ischemic injury

Figure 2 summarizes the neuronal and microglial consequences of crista disruption examined in this section.

**FIGURE 2:**
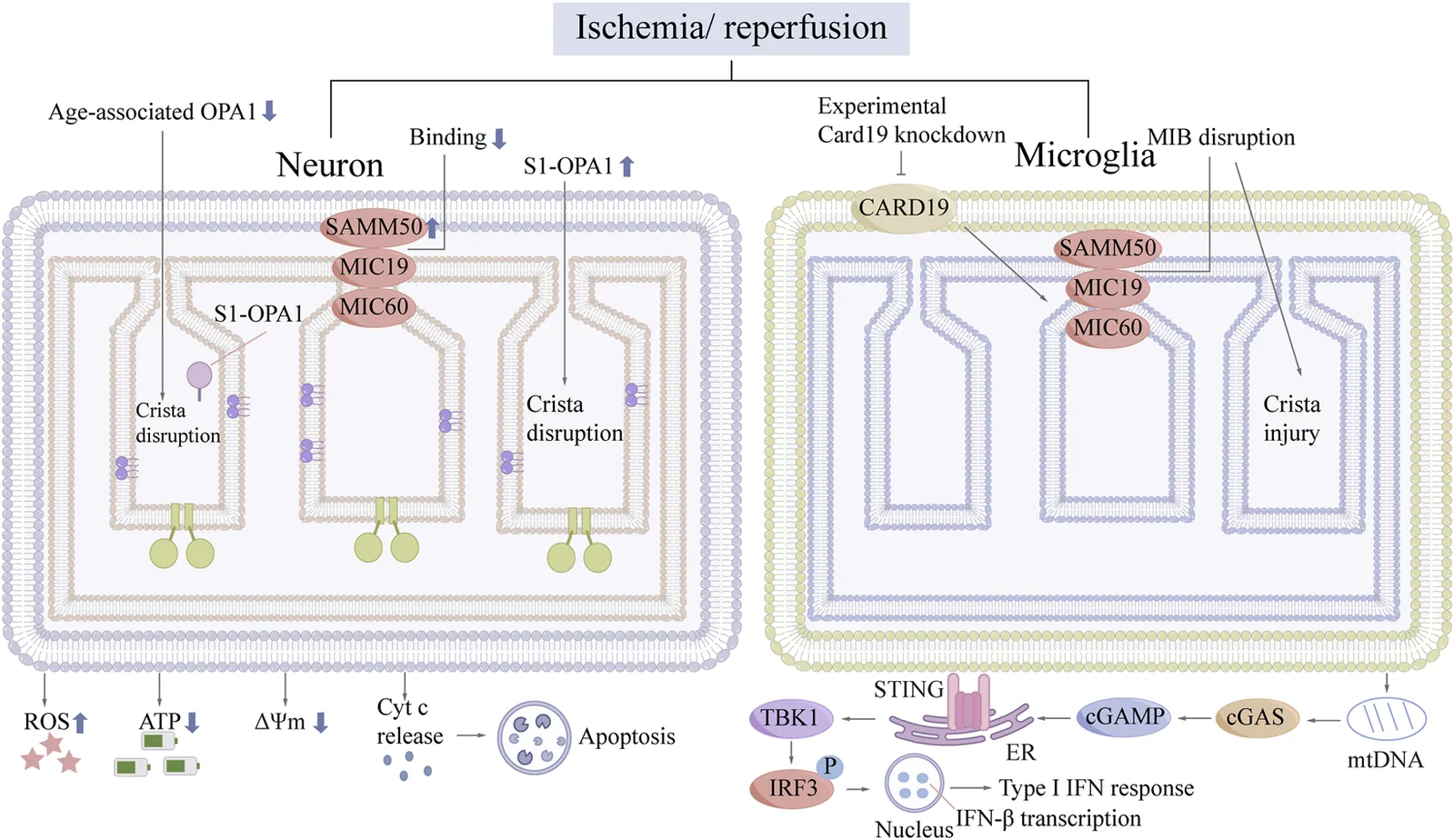
Cristae disruption and associated neuronal and microglial responses in ischemic brain injury. The schematic integrates findings from distinct experimental models of cerebral ischemia and reperfusion. The microglial panel depicts experimentally imposed Card19 knockdown, whereas endogenous CARD19 expression increases after MCAO. The downward arrow beside “Experimental Card19 knockdown” identifies the experimental intervention and does not indicate an expression decrease caused by ischemia. In neurons, age-associated OPA1 decline and increased production of S1-cleaved OPA1 can increase vulnerability to crista disruption (Li et al., 2022; Li et al., 2024c; Li et al., 2026a). SAMM50 abundance can increase despite weakened SAMM50–MIC19 binding, indicating that protein abundance and membrane-bridge integrity may change differently (Yin et al., 2022). These structural disturbances accompany increased reactive oxygen species, reduced ATP production, and loss of mitochondrial membrane potential. Cristae remodeling can facilitate cytochrome c mobilization, which, together with outer-membrane permeabilization, promotes its cytosolic release and apoptotic signaling (Frezza et al., 2006). In microglia, experimental Card19 knockdown destabilizes MIB organization, aggravates mitochondrial ultrastructural injury, and increases cytosolic mtDNA and inflammatory signaling (Hu et al., 2026). Cytosolic mtDNA can activate cGAS-dependent cGAMP production and STING signaling, followed by TBK1-mediated IRF3 phosphorylation, IFN-β transcription, and a type I interferon response. Card19 knockdown represents an experimental perturbation; endogenous CARD19 expression was increased after ischemic injury in the cited study. Upward and downward arrows indicate increases and decreases, respectively; connecting arrows indicate the depicted signaling or functional relationships. Abbreviations: ATP, adenosine triphosphate; CARD19, caspase recruitment domain family member 19; cGAMP, cyclic GMP–AMP; cGAS, cyclic GMP–AMP synthase; Cyt c, cytochrome c; ER, endoplasmic reticulum; IFN, interferon; IRF3, interferon regulatory factor 3; MIB, mitochondrial intermembrane bridge; MIC19/60, MICOS subunits 19/60; mtDNA, mitochondrial DNA; OPA1, optic atrophy 1; ROS, reactive oxygen species; S1-OPA1, OPA1 processed at the S1 cleavage site; SAMM50, sorting and assembly machinery component 50; STING, stimulator of interferon genes; TBK1, TANK-binding kinase 1; ΔΨm, mitochondrial membrane potential. P denotes phosphorylation.

### 3.1 Bioenergetic dysfunction

Reoxygenation restores substrate oxidation in tissue whose inner membranes may remain injured. Succinate accumulated in human and mouse brain tissue studied *ex vivo*. In a mouse model of reperfusion after thrombectomy, the ischemic region rapidly consumed this pool after flow restoration. Quantitative and untargeted metabolomics resolved the accumulation, and mass spectrometry imaging localized the retained succinate to the ischemic region. Systemic malonate infusion at reperfusion reduced acute injury in a dose-dependent manner. This intervention targeted the rapid oxidation of the accumulated pool, providing a way to modify the initial metabolic conditions in which damaged membranes resume electron transport (Mottahedin et al., 2023). Structural recovery followed a longer course in a separate model. After transient global cerebral ischemia, diabetic rats had greater mitochondrial swelling and crista disruption than normoglycemic rats. Injury was most pronounced at 5 hours of reperfusion and remained incompletely resolved at 7 days (Wang and Chen, 2025). The different models address metabolism and ultrastructure on distinct time scales.

During ATP synthesis, respiratory complexes I, III, and IV pump protons from the matrix into the crista lumen. Proton return through ATP synthase drives phosphorylation of ADP at its matrix-facing catalytic head. Energetic conditions also vary within individual cristae. Probes targeted to ATP synthase, complex IV, and the matrix detected distinct pH gradients in living mammalian cells. The gradients changed with substrate use and the direction of ATP synthase activity (Rieger et al., 2021). Mouse cells and isolated mitochondria contained an active ATP synthase population alongside an IF1-bound inactive pool, with IF1 supporting oligomer formation (Romero-Carramiñana et al., 2023). The contribution of OPA1 became apparent when cells were supplied with galactose to increase reliance on mitochondrial ATP production. Under this condition, cristae enlarged, ATP synthase oligomers became less stable, and reactive oxygen species (ROS) accumulated. OPA1 overexpression restrained these changes without increasing the measured mitochondrial antioxidant enzymes. Genetic or pharmacological disruption of ATP synthase oligomerization or activity weakened protection during electron transport blockage. These comparisons connect ROS control to ATP synthase assembly and activity under the imposed metabolic stress (Quintana-Cabrera et al., 2021). These local conditions connect the shape of an assembly with its ability to maintain energy production.

Crista architecture and respiratory assembly interact during energetic recovery. Manipulating crista shape changed supercomplex stability and efficiency in experiments that accounted for protein synthesis and outer-membrane permeabilization (Cogliati et al., 2013). Native porcine structures resolved contacts through which proteins and lipids support these assemblies (Zheng et al., 2024). The energetic response remains dependent on the defect. Mice retained respiration despite markedly reduced respirasome abundance, whereas a larger complex I and III assembly improved function during complex III deficiency and limited cardiac ischemic injury (Milenkovic et al., 2023; Liang et al., 2025). In stroke, assembly measurements are most informative when paired with respiration at the same reperfusion interval.

Lipid remodeling can constrain recovery even when ATP synthase dimers remain intact. Increased phospholipid saturation changed crista topology abruptly in engineered yeast, with CL buffering the transition (Venkatraman et al., 2023). Cardiac-specific TAZ deletion altered CL acyl chains, respiratory assemblies, and mitochondrial architecture (Zhu et al., 2021). At the nucleotide carrier, mutations at distinct CL-binding sites separated carrier destabilization from impaired exchange of adenosine diphosphate (ADP) and ATP (Senoo et al., 2024). Restoring total CL may address only part of a lipid defect that also involves acyl composition and protein binding.

Experimental cerebral ischemia links membrane assembly with the regulation of energy production. In rat ischemia-reperfusion and neuronal oxygen-glucose deprivation followed by reoxygenation (OGD/R), SAMM50 abundance increased while its association with MIC19 weakened. Intracerebroventricular lentiviral vectors were used to increase or reduce SAMM50 before occlusion in adult male rats. Overexpression reduced infarction and neuronal death, improved neurobehavioral measures, and preserved mitochondrial structure and function. Knockdown shifted the same outcomes toward greater injury. The loss of SAMM50-MIC19 interaction under OGD/R is relevant because an increase in the amount of an outer-membrane protein can coexist with failure of the bridge it normally supports (Yin et al., 2022). Increasing sirtuin 3 (SIRT3) promoted OPA1 deacetylation and activity in rat and neuronal models. The *in vivo* preparation used 90 min of transient middle cerebral artery occlusion (MCAO), while cultured rat cortical neurons underwent 2 hours of OGD followed by reoxygenation. Co-immunoprecipitation supported SIRT3-OPA1 interaction in the cortex before and after injury. Increasing SIRT3 improved ultrastructure and energy production alongside smaller infarcts, less apoptosis, and better neuromotor performance. OPA1 knockdown and the activity inhibitor MYLS22 each partly attenuated protection. Expression interference and activity inhibition provide complementary evidence that the state of OPA1 contributes to the SIRT3 response (Chen et al., 2024). These findings illustrate how posttranslational regulation can change the functional state of an existing crista organizer alongside the isoform balance discussed in Section 2.2.

Other mitochondrial interventions operate through broader pathways. Postischemic calcium-channel inhibition reduced calcineurin activation and restored CL concentration, membrane potential, and respiratory control ratio in rodents (Bhattacharya et al., 2025). In mouse transient MCAO and neuronal OGD/R, activator of 90-kDa heat shock protein ATPase homolog 1 (AHA1) bound ATP synthase subunit alpha (ATP5A1) and accompanied energetic imbalance and excessive mitophagy. AHA1 small interfering RNA pretreatment reduced injury (Jin et al., 2025). The architectural contribution of the latter interventions remains unresolved.

Recovery draws on the mitochondrial population retained by the cell and, in some settings, on organelles received from neighboring cells. ATP consumption established metabolically distinct mitochondrial subpopulations in live-cell measurements (Ryu et al., 2024). Pulse-chase proteomics identified long-lived crista proteins in mouse brain and heart (Bomba-Warczak et al., 2021), suggesting that existing assemblies may contribute to early repair. In mouse stroke, astrocytic low-density lipoprotein receptor-related protein 1 (LRP1) restrained lactate production and ADP-ribosylation factor 1 lactylation, supporting mitochondrial transfer to neurons (Zhou et al., 2024). Membrane reorganization and intercellular transfer offer different sources of energetic support to surviving neurons.

### 3.2 Neuronal vulnerability and death

OPA1-dependent crista organization regulates the availability of mitochondrial death factors. Foundational genetic experiments separated its control of apoptotic crista remodeling and cytochrome c mobilization from mitochondrial fusion (Frezza et al., 2006). In neuronal OGD/R and mouse cerebral ischemia, stress increased OPA1 cleavage at the S1 site. Overexpressing the processed form aggravated fragmentation, reduced membrane potential and ATP production, and increased superoxide and mitochondrial apoptosis. Electron microscopy detected corresponding crista damage. A lysine-to-alanine substitution at residue 301 disrupted GTPase function and suppressed the additional injury associated with S1-OPA1 expression. The animal experiments introduced neuron-directed viral expression before stroke, allowing the response of the processed protein to be examined during ischemia and reperfusion. This gain-of-function design identifies a harmful activity of the processed isoform under stress (Li et al., 2022). Moderately increased OPA1 expression reduced infarction in a transgenic ischemia model. Measurements in additional tissues linked crista stability to respiration and cytochrome c retention (Varanita et al., 2015). Aging reduces the protein reserve available for this response.

In aged mice with distal MCAO, neuronal *Opa1* deletion worsened crista disruption and infarction. The investigation first identified age-associated downregulation of mitochondrial genes in a mouse brain expression dataset (GSE212336) and followed OPA1 decline in murine and human material. Proteomic analysis associated OPA1 deficiency and ischemia with reduced translocase of inner mitochondrial membrane 8 homolog B (TIMM8B). TIMM8B overexpression restored OPA1 dimerization, preserved ultrastructure, and reduced apoptosis and infarct expansion in aged ischemic mice. Protection was retained in heterozygous *Opa1*-deficient animals and diminished after complete neuronal *Opa1* loss (Li H. et al., 2026). The remaining OPA1 pool influenced the benefit of this upstream intervention.

Calcium handling links crista organization to neuronal survival through both membrane contacts and uptake machinery. Mitochondrial calcium uptake protein 1 (MICU1) interacted with MIC60 and other crista organizers in cells, with an architectural effect separable from channel regulation (Tomar et al., 2023). OPA1 loss altered endoplasmic reticulum contacts and the calcium signal available for mitochondrial uptake in fibroblasts and patient-derived cells (Cartes-Saavedra et al., 2021). In cortical neurons under OGD/R, netrin-1 activated protein kinase B (AKT) and AFG3-like matrix AAA peptidase subunit 2 (AFG3L2). Immunoblotting identified lower accumulation of essential mitochondrial calcium uniporter regulator (EMRE), with relatively small changes in other regulatory subunits. AFG3L2 loss or AKT inhibition increased the 7-kDa EMRE form, while AKT overexpression raised AFG3L2 in netrin-1-depleted neurons. Reduced mitochondrial calcium and ROS accompanied the protective response. Netrin-1 overexpression also reduced infarction in rat cerebral ischemia, and AKT inhibition weakened that benefit (Yang et al., 2023). Calcium uptake also participates in adaptation. Acute mitochondrial calcium uniporter (MCU) inhibition attenuated the protective depolarization response and increased excitatory calcium signaling in a mouse hippocampal ischemic postconditioning preparation (Sasaki et al., 2024). This context dependence is relevant to interventions aimed at limiting calcium loading during reperfusion.

The permeability transition pore is accessible to intervention near reperfusion. In young male mice with transient MCAO, intravenous cyclosporine A given 5 minutes before reperfusion improved calcium retention and OXPHOS and reduced infarction relative to the area at risk. The neurological score difference was nonsignificant (Ong et al., 2025). The study measured functional mitochondrial rescue without assessing cristae, and cyclosporine A has targets beyond the pore. Its results provide a reperfusion-timed comparator for treatments intended to preserve inner-membrane architecture.

Selective turnover changes the membranes and proteins that survive reperfusion. A MitoTimer reporter in primary neurons identified a prominent phase of older or oxidized protein turnover 2 hours after reoxygenation. Inhibiting lon peptidase 1, mitochondrial (LONP1), or removing parkin RBR E3 ubiquitin protein ligase (PRKN) altered this response (Fogo et al., 2025). Live-cell super-resolution imaging in nonischemic systems revealed lysosome-dependent removal of local inner-membrane domains. The membrane herniated through outer-membrane pores formed by voltage-dependent anion channel 1 (VDAC1). Nearby lysosomes engulfed the protruding material with assistance from the endosomal sorting machinery, generating vesicles that lacked outer membrane and matrix contents. The remaining organelle was spared. Oxidative stress increased the frequency of this process, and lysosomal calcium release was required for vesicle formation. This route provides a physical mechanism for removing a local membrane domain while retaining the rest of the mitochondrion (Prashar et al., 2024). Cryo-electron tomography of depolarized parkin-expressing cells captured ATP synthase redistribution and membranes associated with mitophagic engulfment (Rose et al., 2025). Loss of crista material can accompany selective clearance, making the sequence of membrane damage and removal relevant to its interpretation.

The balance between retention and clearance changes during stroke recovery. Proteasomal loss of BCL2 interacting protein 3 like (BNIP3L/NIX) impaired mitophagy in ischemic brain and cortical neurons. Restoring dimerizing BNIP3L reduced injury, and proteasome inhibition lost its benefit in *Bnip3l*-null mice (Wu et al., 2021). Preconditioning linked the E3 ligase seven in absentia homolog 2 (SIAH2) with early clearance followed by peroxisome proliferator-activated receptor gamma coactivator 1-alpha (PGC-1alpha)-associated recovery (Sisalli et al., 2025). At a later stage, extracellular vesicles carrying circular RNA SCMH1 (circSCMH1), administered 24 h after photothrombotic stroke, restrained an excessive kynurenine 3-monooxygenase-linked mitophagy program and improved motor recovery (Wang Y. et al., 2024). Preserving cristae during recovery must remain compatible with the removal of material that the cell can no longer restore.

### 3.3 Danger signaling and neuroinflammation

Crista injury can expose mitochondrial DNA to inflammatory pathways. A mitochondrial genetic screen linked loss of MICOS components, OPA1, ATP synthase, and CL-related proteins to mtDNA release and a type I interferon response (He et al., 2022). DNA must also traverse the surrounding membranes. In apoptosis models, the inner membrane herniated through enlarged outer-membrane pores before mtDNA escaped (Riley et al., 2018). The relative assembly kinetics of BCL2-associated X protein (BAX) and BCL2 antagonist/killer 1 (BAK) affected pore expansion and the timing of release (Cosentino et al., 2022). Prohibitin 1 loss increased cytosolic mtDNA and inflammation in macrophages, with permeability transition and outer-membrane channel oligomerization contributing to escape (Liu et al., 2022). These routes were established outside ischemic brain models, where the source and path of released DNA need to be resolved.

In ischemic neurons, DNA sensing can feed back onto mitochondrial permeability. Rat transient cerebral ischemia and neuronal OGD/R increased cytosolic oxidized mtDNA and activated cyclic GMP-AMP synthase (cGAS) and stimulator of interferon genes (STING). Restricting release attenuated inflammation and neurological injury (Li Q. et al., 2024). In mouse stroke, neuronal interferon-activated gene 204 (IFI204) promoted STING-associated caspase-1 activation and gasdermin D (GSDMD) cleavage. The GSDMD amino-terminal fragment increased mitochondrial permeability and exposed further mtDNA to cytosolic sensing. IFI204 expression in neurons peaked at 24 h after ischemic injury. Neuron-specific *Ifi204* deletion reduced infarction and improved longer-term sensorimotor and cognitive outcomes, while viral re-expression restored the pathological response. Protein pull-down assays supported direct binding between the IFI204 pyrin domain and STING. STING deletion partly reduced the pyroptotic activity of IFI204. The combined perturbations place mitochondrial permeabilization within a feedback loop in which released DNA sustains its own sensing and further membrane damage (Xu et al., 2026). Astrocytes entered a related inflammatory pathway through metabolic dysfunction. Phosphoglycerate dehydrogenase loss impaired respiration and redox function in mouse ischemia and cultured astrocytes. Increased cytosolic double-stranded DNA accompanied an absent in melanoma 2 (AIM2)-associated pyroptotic response (Zhang J. et al., 2026). The preceding crista changes have not been fully defined in these cell-specific pathways.

Microglial studies connect defined crista assemblies with DNA-associated inflammation. CARD19 expression increased in penumbral microglia after MCAO. The increase was an endogenous response to ischemia. Experimental knockdown tested whether this induced regulator restrained injury and should be distinguished from the expression change produced by ischemia itself. Local *Card19* knockdown driven by a transmembrane protein 119 (*Tmem119*)-Cre/ERT2 system disrupted microglial mitochondria and worsened mouse stroke injury. Cultured microglia under OGD/R accumulated more ROS and cytosolic mtDNA. Interaction assays connected CARD19 with the bridge components and detected weakened SAMM50-MIC19 binding under OGD/R. Increasing CARD19 partly restored this association. Electron microscopy and measurements of cytosolic mtDNA and interferon signaling linked the perturbation to both membrane injury and inflammation. Systemic deoxyribonuclease I partly reduced the additional infarction caused by knockdown. This rescue supports a contribution from exposed DNA, with the releasing cell and precise membrane route remaining unresolved (Hu et al., 2026).

A separate intervention acted at ATP synthase organization. In imiquimod-stimulated murine BV2 cells, icariin II (ICS) bound triose phosphate isomerase 1 (TPI1), promoted its association with ATP5MF, and increased ATP synthase dimers. ICS emerged from a 640-compound phenotypic screen in which fluorescence-based candidates were examined with stimulated emission depletion microscopy and electron microscopy. Chemical proteomics nominated TPI1 as a target, and binding was evaluated with surface plasmon resonance and cellular thermal stability assays. The proposed conformational change enhanced recruitment of ATP5MF. Nano-gold electron microscopy and biochemical fractionation supported mitochondrial recruitment of TPI1 during inflammatory stimulation, which increased further with ICS. ATP5MF knockdown weakened the structural response to ICS. Reduced mtDNA exposure and STING signaling accompanied recovery of OXPHOS, and injury and inflammatory outcomes improved in rat stroke experiments. The approach connects a pharmacologically accessible protein interaction with an assembly inside the crista membrane (Zhang X.-W. et al., 2026).

DNA synthesis contributes to the same inflammatory pool. Microglia/macrophage cytidine/uridine monophosphate kinase 2 (CMPK2) knockdown reduced newly synthesized and oxidized mtDNA, NOD-like receptor family pyrin domain containing 3 (NLRP3) activation, and mouse stroke injury (Guan et al., 2024). Membrane retention and DNA production are distinct upstream routes to reduced inflammatory signaling.

After cytosolic DNA sensing, STING engages several downstream responses in ischemic microglia. In mouse MCAO and BV2 OGD/R, mtDNA exposure accompanied interferon regulatory factor 3 (IRF3) and nuclear factor kappa B (NF-κB) signaling. Early STING inhibition reduced edema, infarction, and neurological impairment (Kong et al., 2022). STING also interacted with NLRP3, whose silencing countered injury produced by STING overexpression (Li W. et al., 2024). A direct interaction with microtubule-associated proteins 1A/1B light chain 3 (LC3) connected STING to microglial autophagy and longer-term behavior in another stroke study (Kong et al., 2024). These downstream pathways provide possible points of intervention after mitochondrial DNA has already entered the cytosol.

Microglial state influences the persistence of mitochondrial inflammatory signals. PGC-1alpha gain of function increased unc-51 like autophagy activating kinase 1 (ULK1)-associated mitochondrial clearance and restrained NLRP3 after mouse stroke. Disrupting ULK1 removed the benefit (Han et al., 2021). After distal MCAO, single-cell analysis identified higher interferon alpha inducible protein 27 like 2A (*Ifi27l2a*) expression in aged microglia. Cell culture and human autopsy material also supported induction of the gene during inflammatory or ischemic conditions. Increasing its expression in microglia was sufficient to increase mitochondrial ROS and promote an inflammatory phenotype. Hemizygous deletion reduced acute and chronic brain injury, microgliosis and astrogliosis, and motor deficits. This pathway gives age-associated inflammatory activation a mitochondrial component, although it has not established a specific change in crista geometry (Kim et al., 2025). Aging and clearance capacity may modify the duration of the inflammatory response to membrane injury. Table 1 summarizes experiments that directly manipulate crista regulators in cerebral ischemia.

**TABLE 1:** Experimental evidence linking cristae organization to ischemic brain injury. Studies are compared by target, model, intervention timing, outcomes, and limitations.

Target or pathway	Experimental model	Intervention and timing	Main findings	Key limitations	References
SAMM50	Male rats, 2-h MCAO/R. Neuronal OGD/R	Preischemic lentiviral gain and loss of SAMM50	Overexpression preserved cristae and membrane bridging, reduced infarction, and improved neurological measures. TEM and interaction assays	Pretreatment design. Other SAMM50 functions may contribute to protection	(Yin et al., 2022)
SIRT3-OPA1	Male rats, 90-min MCAO/R. Cortical neurons, 2-h OGD/R	Preischemic SIRT3 delivery. OPA1 knockdown or MYLS22 inhibition	OPA1 deacetylation and activity accompanied structural and energetic recovery. OPA1 interference partly attenuated protection	Multiple SIRT3 substrates. OPA1 regulates dynamics as well as cristae	(Chen et al., 2024)
OPA1 S1 cleavage	Mouse MCAO/R. Primary neuronal OGD/R	S1-OPA1 or GTPase-defective K301A expression. Neuronal AAV 3 weeks before stroke	S1-OPA1 aggravated crista damage, fragmentation, apoptosis, and injury. K301A suppressed these effects	Processed-isoform gain of function. Fusion and architectural effects remain intertwined. Published figure correction	(Li et al., 2022 ; 2024c)
TIMM8B-OPA1	Aged mice, distal MCAO. Neuronal *Opa1* deletion	TIMM8B overexpression in heterozygous and homozygous *Opa1*-deficient mice	Preserved ultrastructure, restored OPA1 dimerization, and reduced apoptosis and infarction. Protection diminished with complete *Opa1* loss	Requires residual OPA1. Pharmacological efficacy after onset remains unestablished	(Li et al., 2026a)
CARD19-MIB	Male mice, MCAO/R. Microglial OGD/R	Local Cre-dependent AAV knockdown 25 days before stroke. Cell overexpression and post-stroke DNase I rescue	Knockdown increased mitochondrial damage, mtDNA exposure, interferon signaling, and infarction. CARD19 supported SAMM50-MIC19 association. DNase I partly rescued injury	Local knockdown. Systemic DNase I leaves the releasing cell and membrane route unresolved	(Hu et al., 2026)
TPI1-ATP5MF	Male rats, transient MCAO. Imiquimod-stimulated BV2 cells	ICS, 20 mg/kg intraperitoneally daily for 7 days. TPI1 and ATP5MF perturbations	ICS promoted TPI1-ATP5MF association and crista recovery, with reduced mtDNA-STING signaling and stroke injury. TEM, STED, dimer, and metabolic assays	First-dose delay and mitochondrial exposure unclear. TPI1 depletion differs from conformational modulation	(Zhang et al., 2026b)

AAV, adeno-associated virus. ATP5MF, ATP, synthase membrane subunit f. CARD19, caspase recruitment domain family member 19. DNase I, deoxyribonuclease I. ICS, icariin II. MCAO/R, middle cerebral artery occlusion followed by reperfusion. MIB, mitochondrial intermembrane bridge; mtDNA, mitochondrial DNA. OGD/R, oxygen-glucose deprivation followed by reoxygenation. OPA1, optic atrophy 1. SAMM50, sorting and assembly machinery component 50. SIRT3, sirtuin 3. STED, stimulated emission depletion; STING, stimulator of interferon genes; TEM, transmission electron microscopy. TIMM8B, translocase of inner mitochondrial membrane 8 homolog B. TPI1, triose phosphate isomerase 1.

### 3.4 Cristae injury across the neurovascular unit

The neuronal and microglial findings also raise questions about endothelial cells and astrocytes within the neurovascular unit. A plausible shared response is that energetic failure and oxidative stress destabilize membrane assemblies, although the affected crista features and their recovery may differ between cell populations. In brain endothelium, mitochondria containing extracellular vesicles restored respiration and supported survival after oxygen and glucose deprivation (Dave et al., 2023). These results connect mitochondrial rescue with protection of the vascular component of the neurovascular unit, but they do not establish that endothelial crista junctions follow the same remodeling sequence as those in neurons. Matched measurements of crista geometry and barrier function would clarify this relationship.

Astrocytic mitochondrial injury has an additional consequence for metabolic support and communication with neurons. PHGDH loss disrupts respiratory and redox function and promotes DNA associated inflammatory signaling after cerebral ischemia (Zhang J. et al., 2026). Astrocytic LRP1 also regulates mitochondrial transfer to neurons during stroke (Zhou et al., 2024). Together, these observations suggest that preserving astrocytic mitochondrial organization could support both cell survival and neuronal recovery. The proposed commonality is membrane vulnerability to energetic and oxidative stress, whereas the functional emphasis differs between endothelial barrier maintenance, astrocytic support and mitochondrial transfer, neuronal survival, and microglial inflammatory regulation. Resolving these differences provides a rationale for treatments that protect several components of the neurovascular unit.

## 4 Targeting cristae remodeling

Figure 3 summarizes the genetic, pharmacological, and vesicle-based interventions discussed in this section.

**FIGURE 3:**
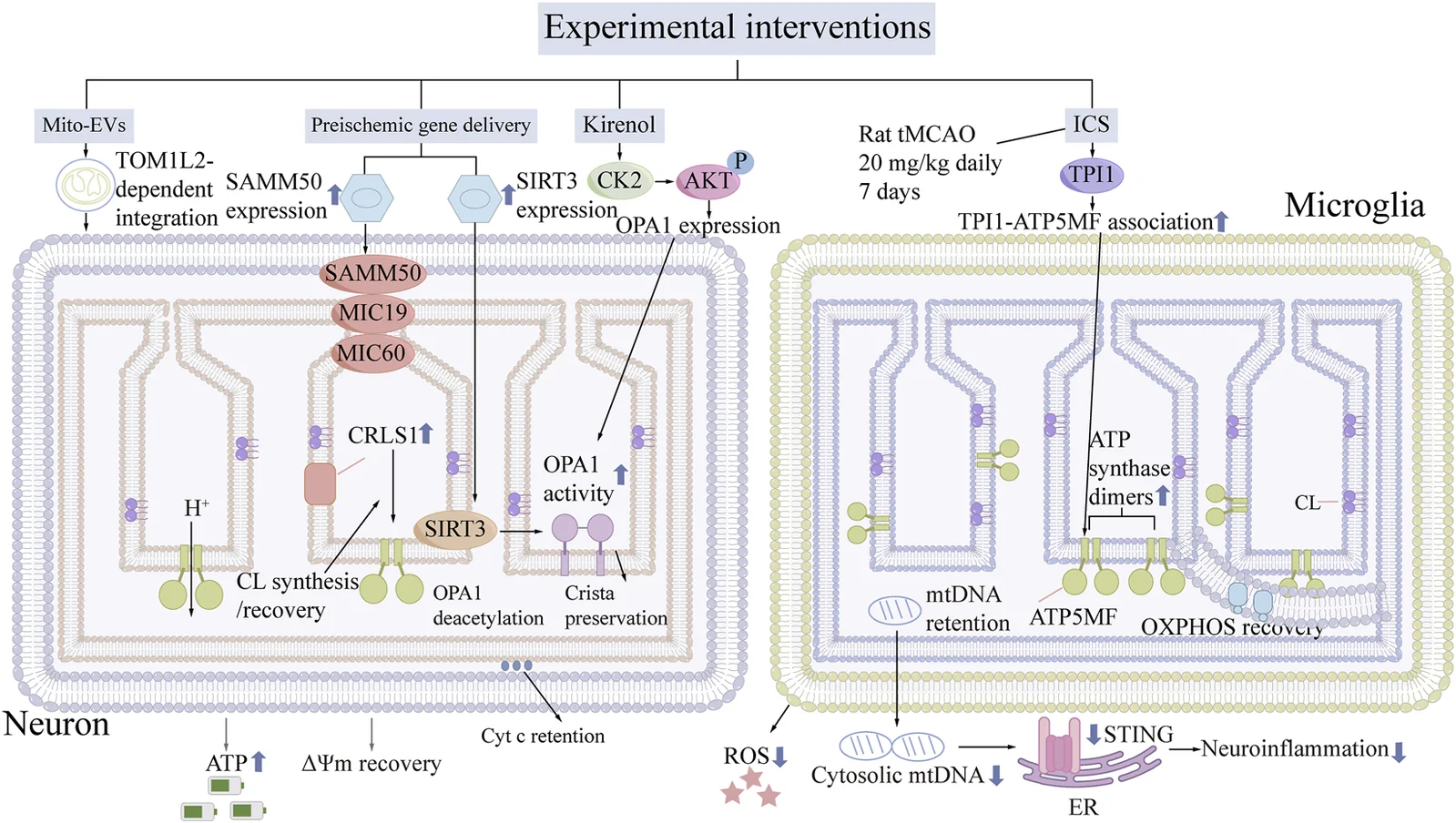
Experimental approaches to restore mitochondrial membrane organization. Preischemic SAMM50 delivery supports neuronal membrane bridging (Yin et al., 2022). SIRT3 promotes OPA1 deacetylation and activity (Chen et al., 2024). Mito-EVs support TOM1L2-dependent integration and CRLS1-associated CL recovery (Li et al., 2026b). Kirenol increases OPA1 expression through CK2/AKT signaling (Zhang et al., 2025). The neuronal branches depict crista preservation, ATP and ΔΨm recovery, and Cyt c retention. In microglia, ICS binds TPI1 and enhances its association with ATP5MF, promoting ATP synthase dimerization. OXPHOS recovery accompanies reduced ROS, cytosolic mtDNA, and STING signaling. The illustrated rat tMCAO regimen is 20 mg/kg daily for 7 days (Zhang et al., 2026b). Connecting arrows indicate functional relationships. Vertical arrows indicate increases or decreases. The STING arrow denotes reduced signaling. P denotes phosphorylation. Abbreviations: AKT, protein kinase B. ATP, adenosine triphosphate. ATP5MF, ATP synthase membrane subunit f. CK2, casein kinase 2. CL, cardiolipin. CRLS1, cardiolipin synthase 1. Cyt c, cytochrome c. ER, endoplasmic reticulum. H^+^, proton. ICS, icariin II. MIC19/60, mitochondrial contact site and cristae organizing system subunits 19/60. Mito-EVs, mitochondria-containing extracellular vesicles. mtDNA, mitochondrial DNA. OPA1, optic atrophy 1. OXPHOS, oxidative phosphorylation. ROS, reactive oxygen species. S1-OPA1, OPA1 processed at the S1 site. SAMM50, sorting and assembly machinery component 50. SIRT3, sirtuin 3. STING, stimulator of interferon genes. tMCAO, transient middle cerebral artery occlusion. TOM1L2, target of Myb1-like 2 membrane trafficking protein. TPI1, triose phosphate isomerase 1. ΔΨm, mitochondrial membrane potential.

### 4.1 Therapeutic strategies and preclinical evidence

Therapeutic development can be organized around stabilizing crista protein assemblies, maintaining their lipid environment, controlling mitochondrial oxidative stress, and delivering functional mitochondrial material. Some interventions directly link a structural response to protection, whereas others provide mitochondrial support that could facilitate subsequent membrane repair. This distinction helps select the structural and functional endpoints needed for each candidate. Recent nanomedicine studies extend the available approaches by improving mitochondrial access and the duration of local exposure. Table 2 compares representative strategies, their relationship to crista biology, and the next step toward application in ischemic stroke.

**TABLE 2:** Therapeutic strategies relevant to crista recovery in ischemic stroke. Evidence is distinguished from proposed applications, with priorities for further development.

Therapeutic strategy	Target and rationale	Evidence and timing	Relationship to cristae	Development priority	References
SAMM50, SIRT3, and TIMM8B modulation	Membrane bridging, OPA1 activity, and OPA1 dimerization	Genetic protection in rodent stroke models, mainly with expression established before injury	Direct ultrastructural responses with pathway perturbation supporting target relevance	Achieve rapid target engagement after stroke onset.	(Yin et al., 2022; Chen et al., 2024; Li et al., 2026a )
ICS and kirenol	TPI1 association with ATP5MF or upstream signaling that supports OPA1	Pharmacological protection in experimental stroke. ICS was given daily for 7 days	ICS links ATP synthase dimerization with crista recovery. Kirenol supports OPA1 related mitochondrial function	Relate brain exposure and dose timing to assembly engagement	( Zhang et al., 2026b; Zhang et al., 2025 )
Cardiolipin directed treatment	Support the lipid environment of crista shaping and respiratory proteins	Elamipretide has clinical experience and accelerated approval in Barth syndrome, informing further therapeutic development	Mechanistic rationale for membrane stabilization, with the stroke structural response to be measured	Establish acute brain exposure, lipid engagement, and membrane recovery	( Karaa et al., 2023; U.S. Food and Drug Administration, 2025 )
Mitochondria targeted ceria nanoenzymes	Combine ROS control with targeted drug carriage	Neuroprotective mitochondrial nanomedicine in experimental ischemic stroke	A candidate for linking mitochondrial antioxidant protection with quantitative crista recovery	Pair catalytic activity and mitochondrial exposure with quantitative crista imaging	(Liao et al., 2024)
Mitochondria containing extracellular vesicles	Supply functional mitochondrial material and support lipid and respiratory recovery	Protection in neuronal and endothelial injury models and experimental stroke	Organelle delivery can support membrane recovery alongside host mitochondrial function	Standardize mitochondrial cargo, potency, recipient uptake, and dose timing	( Dave et al., 2023; Li et al., 2026b )
Sequential brain and mitochondrial delivery	Combine tissue access, cell targeting, and active cargo release near mitochondria	Experimental stroke studies include vascular and intranasal formulations	A platform for reaching crista targets within the intended cells	Measure intact cargo and structural engagement beyond carrier colocalization	( Wang et al., 2024a; Yin et al., 2026; Liao et al., 2025 )
Vascular retention and sustained release	Extend local exposure through ligand triggered anchoring or controlled release	Anchoring was demonstrated in a thrombosis model. Sustained brain delivery is reviewed across neurological diseases	Delivery principles that may inform future crista directed formulations	Align release with reperfusion and recovery while confirming brain and mitochondrial access	( Liao et al., 2026b; Liao et al., 2026a )
Reperfusion timed metabolic and permeability modulation	Malonate limits succinate oxidation. Cyclosporine A modulates permeability transition	Mitochondrial protection in mice with dosing near reperfusion	Functional comparators for evaluating structural protection during reperfusion	Test membrane architecture alongside metabolism and lasting functional recovery	( Lee et al., 2025; Ong et al., 2025 )
Recovery phase quality control and inflammation	Regulate mitochondrial turnover and DNA associated inflammatory signaling	circSCMH1 vesicles and delayed cGAS and STING inhibition support recovery in experimental stroke	Complementary support after the acute oxidative phase	Compare staged treatment with each intervention alone at matched exposures	( Wang et al., 2024b; Maimaiti et al., 2024 )

AAV, adeno associated virus. ATP5MF, ATP, synthase membrane subunit f. CL, cardiolipin; cGAS, cyclic GMP AMP, synthase; ICS, icariin II. MICOS, mitochondrial contact site and cristae organizing system. OPA1, optic atrophy 1. ROS, reactive oxygen species. SAMM50, sorting and assembly machinery component 50. SIRT3, sirtuin 3. STING, stimulator of interferon genes. TIMM8B, translocase of inner mitochondrial membrane 8 homolog B. TPI1, triose phosphate isomerase 1.

#### 4.1.1 Modulating crista protein assemblies

The clearest genetic evidence for protection comes from interventions established before ischemia. Viral SAMM50 expression established before occlusion preserved mitochondrial ultrastructure after ischemia-reperfusion in rats (Yin et al., 2022). Adeno-associated virus (AAV)-mediated SIRT3 delivery also preceded ischemia and identified OPA1 dependence (Chen et al., 2024). These approaches show that increasing a regulator can influence the subsequent injury response. Therapeutic use after onset adds a requirement for the damaged cell to rebuild or stabilize the assembly within the remaining treatment window.

The CARD19 and ICS studies address different stages of this development. *Tmem119*-Cre/ERT2-dependent *Card19* knockdown preceded MCAO and tested the contribution of the endogenous regulator (Hu et al., 2026). ICS was tested pharmacologically in a transient occlusion model. Male rats received 20 mg/kg intraperitoneally each day for 7 days. In a separate photothrombotic mouse experiment, ionized calcium-binding adaptor molecule 1 (Iba1)-promoter AAV-shRNA delivery had approximately 60% reported microglial specificity. TPI1 depletion itself reproduced part of the neuroprotective response in this supplementary experiment (Zhang X.-W. et al., 2026). The intended manipulation is a binding-induced change in the TPI1 interaction network. TPI1 depletion and binding-induced conformational modulation have different effects on respiration and crista organization. Their overlapping effects on injury complicate a simple interpretation in which increasing TPI1 activity is uniformly protective. Measurements of the TPI1-ATP5MF association alongside catalytic activity would help define the response at a given drug concentration. The interval to the first dose and free drug exposure within microglial mitochondria were not established. These gaps limit the treatment window and degree of cell selectivity that can be inferred from the reported protection.

Upstream signaling and RNA regulation offer routes to modulate OPA1. Kirenol improved membrane potential, ATP production, and neurological outcomes in experimental cerebral ischemia. Casein kinase 2 and AKT signaling accompanied increased OPA1, while pathway inhibition or OPA1 reduction weakened protection (Zhang et al., 2025). The architectural contribution remained intertwined with other mitochondrial effects. Antagonizing OPA1-repressing microRNAs raised OPA1 and improved myopathy phenotypes in mice (Djalalvandi et al., 2025). Processing could modify the response to either route, since preventing stress-induced OPA1 cleavage worsened mouse mitochondrial cardiomyopathy (Ahola et al., 2024). A candidate that raises total OPA1 may change the balance and activity of its isoforms at the same time.

The pharmacological opportunity is to preserve useful OPA1 and MICOS assemblies while retaining the processing required for adaptation. The SAMM50, SIRT3, and TIMM8B experiments identify complementary routes to membrane bridging, OPA1 activity, and OPA1 dimerization. Their value for drug development lies in defining measurable interactions that can be tested after stroke onset. For ICS and kirenol, measuring assembly engagement at concentrations achieved in the brain would help connect the protective response with a feasible dosing regimen. Combining ultrastructural measurements with selective pathway perturbation would further guide the choice of compounds for subsequent studies.

#### 4.1.2 Protecting cardiolipin and controlling oxidative stress

Cardiolipin directed approaches offer a way to support the membrane environment shared by OPA1, MICOS, respiratory complexes, and ATP synthase. Elamipretide is a cardiolipin binding peptide with clinical experience in mitochondrial disease (U.S. Food and Drug Administration, 2025). This provides a practical starting point for evaluating dosing, systemic tolerability, and formulation requirements. In ischemic stroke, its relevance to crista repair would be established by relating brain exposure to cardiolipin composition, membrane organization, and respiratory recovery. Lipid directed treatment could also complement protein directed interventions when membrane damage persists despite adequate amounts of crista organizers.

Mitochondrial antioxidant nanomedicine adds a pharmacological strategy that can address the oxidative environment in which crista assemblies recover. Liao and colleagues developed a ceria nanoenzyme system combining mitochondrial targeting, catalytic control of ROS, and drug carriage for experimental ischemic stroke (Liao et al., 2024). The study is valuable because it connects delivery to injured neuronal mitochondria with treatment of reperfusion associated oxidative injury. In the context of crista biology, this strategy could help protect the lipid and protein environment needed for membrane recovery. Pairing its mitochondrial effects with quantitative crista imaging would establish how structural preservation contributes to the reported protection.

#### 4.1.3 Mitochondrial delivery and control of local exposure

Lipid recovery and organelle delivery offer additional support for rebuilding membrane assemblies. Calcium-channel inhibition restored CL concentration and respiratory measurements after rodent cerebral ischemia, although the intervening lipid pathway remained unresolved (Bhattacharya et al., 2025). Mitochondria-containing vesicles from human umbilical cord mesenchymal stromal cells improved neuronal mitochondrial measurements and rat stroke outcomes. Donor-cell depletion of target of Myb1-like 2 membrane trafficking protein (TOM1L2) weakened integration and energetic rescue. Recipient cardiolipin synthase 1 (CRLS1) was linked to lipid recovery (Li Z. et al., 2026). Larger extracellular vesicles supported respiration in oxygen-glucose-deprived brain endothelial cells, with limited evidence on lasting efficacy from the accompanying mouse pilot (Dave et al., 2023). Reproducible cargo composition and uptake into the intended cells remain central to evaluating this delivery approach.

A delivery system must provide access to the mitochondrial target after reaching the injured brain. Sequential targeting to injured tissue and neuronal mitochondria improved oxidative and inflammatory measurements in experimental cerebral ischemia, using a formulation that also carried antioxidant activity (Wang S. et al., 2024). An intranasal nanolamellar formulation increased brain-associated signal and mitochondrial colocalization and improved recovery in rats. It contained several active components with immune effects (Yin et al., 2026). Imaging establishes carrier localization. Quantifying intact active cargo at the target would relate that localization to the exposure needed for a structural response.

A broader framework for these delivery approaches comes from the integration of mitochondrial heterogeneity, disease context, and targeting technology in brain disorders (Liao et al., 2025). Access to the brain and access to the intended mitochondrial compartment are distinct stages. An effective formulation should therefore combine delivery to the relevant neural or vascular cell with release of active cargo near its mitochondrial target. For crista directed agents, this framework helps connect tissue targeting with a specific membrane response and supports comparison of neuronal, microglial, astrocytic, and endothelial treatment effects.

Local retention offers another way to improve exposure during reperfusion. Ligand triggered topology switching converted circulating nanospheres into an adhesive nanofibrillar network after integrin recognition and supported durable anchoring in a thrombosis model (Liao et al., 2026b). Its contribution here is a delivery principle for maintaining local exposure under vascular flow. For crista directed payloads, this principle suggests coupling local anchoring with brain distribution and mitochondrial access. The thrombosis study provides a useful foundation for designing delivery under the vascular conditions encountered at reperfusion.

Sustained delivery may extend mitochondrial support beyond the first oxidative burst. A recent review describes diffusion, degradation, and stimulus responsive release mechanisms for long acting brain nanomedicine (Liao et al., 2026a). For stroke, the useful release profile should follow the changing injury response. Rapid availability may be needed near reperfusion, followed by a controlled maintenance phase while inflammation and mitochondrial recovery continue. Measuring release kinetics alongside brain exposure and crista recovery would help select a formulation whose duration matches the period during which the membrane target remains responsive.

#### 4.1.4 Treatment timing and staged interventions

Reperfusion supplies a defined time point for mitochondrial drug delivery. In mice, intra-arterial acidified malonate at reperfusion reduced 24-h magnetic resonance infarct volume and improved neurological scores. The mouse occlusion model was adapted to permit local arterial infusion as the ischemic territory was reperfused. Acidified disodium malonate at 160 mg/kg and pH 6 reduced infarct volume by approximately 60% at the 24-h assessment. Local administration creates a practical connection between a mitochondrial intervention and the vascular procedure used to restore flow (Lee et al., 2025). Intravenous cyclosporine A 5 minutes before reperfusion improved calcium retention and OXPHOS in young male mice, with a nonsignificant difference in neurological score (Ong et al., 2025). These regimens provide functional benchmarks at early reoxygenation. Neither experiment established that crista preservation mediated the benefit.

Later dosing addresses mitochondrial clearance and inflammation during recovery. Proteasome inhibition produced a BNIP3L-dependent benefit at onset and at delayed experimental time points (Wu et al., 2021). Vesicles carrying circSCMH1, given 24 h after photothrombotic stroke, improved motor recovery while restraining excessive mitophagy (Wang Y. et al., 2024). cGAS-STING inhibition begun 3 days after mouse stroke improved remyelination and longer-term function (Maimaiti et al., 2024). The different schedules identify opportunities for testing whether crista stabilization remains useful after the acute metabolic disturbance has subsided.

These schedules suggest a staged therapeutic perspective. An early intervention could moderate substrate driven oxidative stress or calcium dependent permeability during reperfusion, while a subsequent intervention supports membrane assembly, lipid recovery, or mitochondrial quality control. This is a testable strategy rather than an established treatment regimen. Its evaluation should compare the combination with each component alone at matched exposures and determine whether preserved cristae accompany sustained neurological recovery. Endothelial protection and astrocytic mitochondrial support may complement neuronal rescue by maintaining the surrounding tissue environment. The most informative sequence will therefore depend on the cell population engaged, the speed of target modulation, and the reperfusion setting.

### 4.2 Translational challenges

#### 4.2.1 Treatment window and target exposure

Age and metabolic disease can change the reserve available for membrane repair. OPA1 depletion increased neuronal vulnerability in aged mice with distal MCAO (Li H. et al., 2026), and diabetes intensified ultrastructural injury after global cerebral ischemia in rats (Wang and Chen, 2025). Post-onset dosing in these backgrounds would test whether the intended assembly remains recoverable and whether comorbidity narrows the useful treatment window.

Target engagement has a regional and cellular context. Spatial and single-cell profiling resolved distinct peri-infarct cellular programs in mouse ischemia (Han et al., 2024). Human and mouse astrocytes differed in susceptibility to oxidative stress and responses to hypoxic repair (Li et al., 2021). Injury severity also changed with experimental state. A minimally invasive thrombotic protocol produced larger lesions in awake than anesthetized mice (Marks et al., 2025). Localizing exposure and structural recovery to the affected cells would make whole-tissue mitochondrial measurements easier to interpret. Human cell models could assess whether the same response is retained across species.

Treatment timing should be defined by the biology of the candidate and the clinical setting in which it would be used. Interventions intended to suppress the initial mitochondrial oxidative burst are most naturally evaluated around recanalization, whereas agents supporting assembly repair or sustained mitochondrial transfer may retain value during later recovery. Experimental dose delay curves can relate these opportunities to residual tissue viability and target engagement. Testing alongside reperfusion therapy, with prespecified dosing before or after recanalization, would make this relationship more useful for clinical development. Comparisons across age, sex, and metabolic risk can identify the populations most likely to retain a responsive membrane target.

Delivery assessment should connect the administered dose with intact active cargo in the affected brain cells and their mitochondrial compartment. Regional drug measurements, cell resolved uptake, and assays of the intended protein interaction or lipid response provide complementary information. For formulations whose mitochondrial uptake depends on membrane potential, uptake during depolarization should be measured directly. A positive carrier signal should be interpreted together with cargo release and functional target engagement. These measurements can guide formulation selection and identify an exposure range that supports membrane recovery while preserving physiological mitochondrial activity.

#### 4.2.2 Target engagement and three dimensional structural validation

Structural endpoints should describe the membrane feature engaged by treatment. Three-dimensional cryo-electron tomography showed that OPA1 state altered crista stacking, packing, and organelle shape in different combinations (Fry et al., 2024). Prespecified regions and separate measures of junctions, lumen width, and lamellar organization would distinguish these responses. Respiration and survival measured in matched biological samples would relate them to function. The same design makes negative results interpretable. Treatment could restore the number of junctions while leaving lamellar packing or lumen width abnormal, and these responses should remain separate in the analysis. Repeated sampling at early and later reperfusion intervals would test whether structural recovery precedes improvement in respiration or accompanies it. Imaging and functional measurements should be drawn from comparable regions so that a change in the sampled cell population is not mistaken for repair within cells. When many mitochondria are imaged, the animal or independent culture remains the biological unit of inference. Table 3 summarizes methods and controls for evaluating cristae-dependent protection.

**TABLE 3:** Experimental approaches for evaluating cristae-dependent neuroprotection. The proposed methods and controls draw on structural, mechanistic, and intervention studies.

Assessment domain	Methods and endpoints	Controls and comparisons	Key limitations	References
Crista architecture	TEM and three-dimensional tomography. Junctions, lumen width, and lamellar organization	Matched cell type, region, and reperfusion interval. Blinded sampling and animal-level or culture-level analysis	Two-dimensional sections incompletely represent topology. Multiple mitochondria from one sample remain nested observations	(Fry et al., 2024; Zheng et al., 2024)
Structural dynamics	Live super resolution imaging paired with membrane potential and respiration. Depth resolved three dimensional reconstruction when validated	Matched section thickness and imaging depth. Unlabeled controls, background and resolution calibration, probe loading and illumination controls. Verification by electron tomography or correlative imaging	High tissue background and axial blur can merge adjacent cristae. Probe behavior and segmentation affect measurements. Report unresolved features and reconstruction uncertainty	(Prashar et al., 2024; Zhang et al., 2026b; Hao et al., 2021; Papereux et al., 2025 )
Assembly and lipid composition	Native-complex assays, protein interactions, and CL species or acyl composition	Loss of function with rescue. Separate protein abundance from oligomerization and lipid composition	Association alone leaves necessity unresolved. Preserved dimers may coexist with impaired curvature	(Galber et al., 2021; Venkatraman et al., 2023)
Structural contribution to protection	Architecture and injury outcomes alongside fusion or catalytic activity	Function-selective mutants where available. Protection tested during disruption of the structural response	A regulator can alter several functions. Parallel structural and energetic recovery leaves mediation unresolved	(Frezza et al., 2006; Galber et al., 2021)
Death-factor and DNA release	Cytochrome c fractionation, cytosolic mtDNA, and pore imaging	Fraction-purity and mitochondrial-origin controls. Release paired with pathway inhibition or rescue	Cytosolic DNA measurements are vulnerable to contamination. Systemic DNA clearance leaves the releasing cell and route unresolved	(Riley et al., 2018; Hu et al., 2026)
Treatment after stroke onset	Timed dosing, cell-specific structural engagement, and lasting behavioral recovery	Age, both sexes, and metabolic risk. Permanent versus reperfused injury. Brain exposure related to target response	Preischemic expression establishes target relevance. Drug benefit at reperfusion still needs evidence of crista dependence	(Lee et al., 2025; Li et al., 2026a)

ATP, adenosine triphosphate; CL, cardiolipin; mtDNA, mitochondrial DNA. TEM, transmission electron microscopy.

Accurate three dimensional quantification presents a further challenge in thick brain sections and ischemic regions with high background fluorescence. Tissue autofluorescence, scattering, nonspecific labeling, and signal from overlapping mitochondria can reduce membrane contrast, while depth dependent aberrations impair axial resolution. Adjacent cristae may consequently appear merged, and segmentation thresholds may change estimates of lumen width, membrane surface area, or junction density. Fluorescent probes that depend on membrane potential can also change their distribution during ischemia, making signal intensity an imperfect measure of membrane architecture. Adaptive optics can improve three dimensional nanoscopy in thick specimens (Hao et al., 2021), providing a technical basis for extending structural measurements into brain tissue.

Reliable quantification should pair optical improvement with validation of the reconstructed membrane features. Matched section thickness, anatomical region, and imaging depth can be combined with unlabeled controls, background measurements, and calibration of lateral and axial resolution. Spectral separation, optical sectioning, and validated deconvolution may improve contrast, with structural preservation checked after fixation or clearing. DeepCristae illustrates how computational restoration can assist crista visualization, although its training on two dimensional STED images calls for validation when applied to ischemic tissue (Papereux et al., 2025). Representative fluorescence reconstructions should be checked against electron tomography or correlative imaging in matched regions. Reporting unresolved structures, segmentation uncertainty, and biological replication would make estimates of crista surface density, junction number, and lumen width more reproducible.

#### 4.2.3 Product consistency and safety assessment

Translation also benefits from defining the properties that make a carrier or vesicle product reproducible. For nanoenzymes, these include particle size, surface chemistry, catalytic activity, cargo loading, and release kinetics. For mitochondria containing vesicles, donor cell identity, mitochondrial content, respiratory competence, and uptake by recipient cells are relevant measures. Batch comparisons should connect these product attributes with the ability to produce the intended membrane response. Stability during storage and preparation for administration should be incorporated into the same assessment, so that the material used in efficacy studies can be reproduced for subsequent development.

Safety evaluation should follow the delivery route and treatment duration. Vascularly retained formulations can be examined for effects on perfusion, coagulation, and compatibility with reperfusion therapy. Intranasal formulations can be evaluated for local tolerability and consistency of brain delivery. For repeated dosing or organelle delivery, biodistribution, clearance, and inflammatory responses to mitochondrial material merit targeted assessment. These studies are most useful when performed at exposures associated with mitochondrial target engagement. They can guide dose selection and support the development of a delivery strategy appropriate to the intended acute or recovery phase of stroke treatment.

#### 4.2.4 Clinical development and functional outcomes

Clinical CL-directed treatment provides experience with systemic dosing in diseases outside stroke. Elamipretide did not improve either primary functional endpoint in MMPOWER-3, a randomized trial of 218 participants with primary mitochondrial myopathy (Karaa et al., 2023). An open-label Barth syndrome extension reported longer-term functional and CL-related changes without a concurrent randomized comparator (Thompson et al., 2024). In September 2025, the U.S. Food and Drug Administration granted accelerated approval to elamipretide for improving muscle strength in patients with Barth syndrome weighing at least 30 kg, with a confirmatory trial required (U.S. Food and Drug Administration, 2025). This regulatory experience supports the clinical development of cardiolipin directed treatment, while the distinct requirements of acute stroke still call for direct assessment of brain exposure and benefit in reperfused tissue. Acute cerebral ischemia adds questions about free brain exposure, uptake by the affected cells, and the speed of the structural response. These measurements would connect a systemic dose with engagement of the brain target.

Clinical cytoprotection trials place treatment efficacy within a specific reperfusion setting. Nerinetide did not improve 90-day functional independence among 850 thrombectomy patients without prior thrombolysis in ESCAPE-NEXT (Hill et al., 2025). A 200-participant phase II trial of edaravone dexborneol after successful thrombectomy did not meet its functional primary endpoint (Chen et al., 2025). TASTE-2 enrolled patients within 24 h of symptom onset who had anterior-circulation large-vessel occlusion and planned thrombectomy. Edaravone dexborneol was started before the procedure and continued twice daily for 10–14 days. Ninety-day independence occurred in 55.0% versus 49.6% of patients, with a risk ratio of 1.11 and a 95% confidence interval of 1.00–1.23. The absolute difference was 5.4 percentage points. A larger apparent response in the admission mismatch subgroup supports further evaluation of patient selection (Wang et al., 2026). TASTE-SL found benefit in a differently selected acute stroke population (Fu et al., 2024). For a mitochondrial candidate, patient selection should be linked to the expected persistence of a modifiable target and the timing of reperfusion.

The decisive experimental step is to identify which response mediates protection. A regulator may alter respiration, death-factor release, and DNA-associated inflammation together. Selective mutants and rescue experiments can test dependence on the structural function. A useful comparison would retain a regulator’s catalytic or fusion activity while weakening its membrane-organizing interaction, or restore that interaction in a deficient background. Injury measured alongside the structural response could then distinguish target engagement from the response required for benefit. Pharmacological work would add concentration and timing to this comparison, making it possible to identify exposure that changes architecture before broad metabolic inhibition appears. Post-onset dosing can then assess whether that response remains accessible in aged animals and in reperfused or nonreperfused tissue. Circulating markers would be useful only once they can be related to target engagement in the affected brain cells.

An initial clinical program could therefore prioritize a defined reperfusion population, an achievable dosing schedule, and a pharmacodynamic measure linked to the proposed mitochondrial response. Early studies would establish tolerability and exposure, followed by evaluation of functional recovery with imaging and molecular measures as supportive endpoints. Standardizing recanalization status and concurrent treatment would help interpret any added benefit. This development pathway connects the mechanistic evidence with a concrete therapeutic objective and provides a framework for selecting crista directed candidates for clinical testing.

## 5 Discussion and concluding remarks

Crista remodeling connects inner-membrane organization with the energetic and inflammatory responses to ischemia. MICOS and MIB maintain junctions and membrane contacts, while OPA1 processing, ATP synthase geometry, and CL composition alter the properties of the crista membrane. Stroke experiments have begun to link specific regulators with structural recovery and reduced injury. The evidence is strongest where perturbation of the proposed pathway changes both architecture and the protective response.

Treatment after onset remains the main test of clinical relevance. Preischemic gene expression identifies assemblies that influence vulnerability, but rebuilding them in an injured neuron or microglial cell may require a different intervention. Pharmacological studies now offer accessible protein interactions and reperfusion-timed regimens. Their value will depend on the relationship between exposure, the speed of membrane recovery, and the subsequent course of clearance and repair. The expanded therapeutic framework combines protein and lipid directed interventions with mitochondrial delivery and sustained local exposure. A staged approach linking acute mitochondrial protection with subsequent membrane recovery provides a practical hypothesis for testing adjunctive treatment alongside reperfusion therapy.

Future work should follow the structural response within defined cell populations and relate it to sustained neurological recovery. The same measurements can establish when an assembly remains modifiable and when protection is lost despite its restoration. This approach would help select candidates whose effects on cristae support lasting benefit after stroke.
